# Does universal nasal/skin decolonization in nursing homes affect risk factors for MRSA carriage?

**DOI:** 10.1017/ash.2023.332

**Published:** 2023-09-29

**Authors:** Gabrielle Gussin, Raveena D. Singh, Thomas Tjoa, James A. McKinnell, Loren Miller, Susan Huang

## Abstract

**Background:** A regional decolonization intervention (SHIELD-OC) involving universal chlorhexidine for routine bathing and 5 days of twice-daily nasal iodophor every other week in nursing homes (NHs) recently demonstrated marked reductions in multidrug-resistant organisms, all-cause hospitalizations, and infection-related hospitalizations in Orange County, California. Specific to methicillin-resistant *Staphylococcus aureus* (MRSA), NH prevalence (nares, skin, or perirectal) decreased from 43% to 29%. **Methods:** We conducted a retrospective cohort study evaluating the impact of decolonization on factors associated with MRSA carriage. The cohort included residents from 18 SHIELD-OC NHs who were sampled for MRSA using nares, axilla, groin, and perirectal cultures. A point-prevalence survey was conducted in 2016–2017 (before decolonization, 50 randomly sampled residents per NH) and in 2018–2019 (decolonization, all residents sampled). Resident characteristics were obtained from their most proximal admission, quarterly, and/or discharge assessment using data mandated for NH reporting (CMS minimum data set), and included demographics, medical devices, comorbidities (including Alzheimer’s disease and related dementias or ADRD), and mobility and hygiene needs. We used generalized-linear mixed models stratified by decolonization and clustered by NH to identify differences in factors associated with MRSA carriage. **Results:** Of the 2,351NH residents, 2,255 (96%) had characteristics available in the CMS data set. Of the 2,255 residents included, 774 (34%) were MRSA carriers. Before decolonization, medical devices (OR, 2.5), limited mobility (OR, 1.6), and diabetes (OR, 1.4) were significantly associated with MRSA carriage in an adjusted model (Table). During decolonization, these effects were mitigated (medical device OR, 2.5–1.1; diabetes OR, 1.4–0.9) and were no longer significantly associated with MRSA carriage. Male sex appeared to have more of an effect in the decolonization phase (OR, 1.3–1.6), but limited mobility remained stable (OR, 1.6–1.7). Several variables were collinear. Presence of a medical device was collinear with postacute stays (<100 days) and Medicaid insurance. Limited mobility was associated with limited ability for hygienic self-care. ADRD was collinear with age. Final adjusted models accounted for medical devices, limited mobility, diabetes, ADRD, cancer, sex, and ethnicity. **Conclusions:** In a large interventional cohort of 18 NHs, factors associated with MRSA carriage changed after adoption of universal decolonization. Specifically, the increased risk of MRSA associated with medical devices and diabetes were substantially mitigated by decolonization, suggesting that these risks are modifiable. These long-term care findings are consistent with clinical trials showing reductions in MRSA carriage after implementing chlorhexidine bathing in ICUs and in non-ICU patients with devices. The ability of decolonization to attenuate the risk of MRSA carriage among diabetics or other potential high-risk groups deserves further study.

**Figure:**
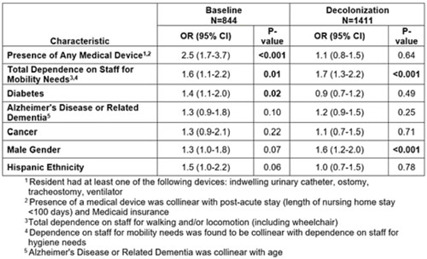

**Disclosures:** None

